# Research on the Factors Affecting the Growth of Large-Size Monolayer MoS_2_ by APCVD

**DOI:** 10.3390/ma11122562

**Published:** 2018-12-17

**Authors:** Tao Han, Hongxia Liu, Shulong Wang, Wei Li, Shupeng Chen, Xiaoli Yang, Ming Cai

**Affiliations:** 1 Key Laboratory for Wide-Band Gap Semiconductor Materials and Devices of Education, School of Microelectronics, Xidian University, Xi’an 710071, China; 15639119745@163.com (T.H.); li20101467@163.com (W.L.); chenshupeng999@126.com (S.C.); cm9999787@163.com (M.C.); 2 The School of Mathematics and Statistics, Xidian University, Xi’an 710071, China; xiaoyangxiaoli163@163.com

**Keywords:** monolayer MoS_2_, APCVD, Raman spectrum, growth condition, PL spectrum

## Abstract

The transition-metal chalcogenides (TMDs) are gaining increased attention from many scientists recently. Monolayer MoS_2_ is an emerging layered TMD material with many excellent physical and electrical properties. It can be widely used in catalysis, transistors, optoelectronics and integrated circuits. Here, the large-sized monolayer MoS_2_ is grown on the silicon substrate with a 285-nm-thick oxide layer by atmospheric pressure chemical vapor deposition (APCVD) of sulfurized molybdenum trioxide. This method is simple and it does not require vacuum treatment. In addition, the effects of growth conditions, such as sulfur source, molybdenum source, growth temperature, and argon flow rate on the quality and area of MoS_2_ are further studied systematically. These analysis results help to master the morphology and optical properties of monolayer MoS_2_. The high quality, excellent performance, and large-size monolayer MoS_2_ under the optimal growth condition is characterized by optical microscopy, AFM, XPS, photoluminescence, and Raman spectroscopy. The Raman spectrum and PL mapping show that the grown MoS_2_ is a uniform triangular monolayer with a side length of 100 μm, which can pave the way for the applications of photodetectors and transistors.

## 1. Introduction

In recent years, two-dimensional materials have been a hot topic in Nano-electronic research [1,2,3]. Many scientists have conducted extensive research on graphene with ultra-thin, ultra-strong and super-conductive properties. The pristine graphene has no band gap, so it cannot be used in the logic circuits. To compensate for the lack of band gap in graphene, researchers have begun to focus on the monolayer MoS_2_ with its graphene-like structure [4]. Monolayer MoS_2_ has a sandwich layered structure, the upper and lower layers are hexagonal planes composed of sulfur atoms, and the middle is a metal molybdenum atomic layer [5]. The atoms in monolayer MoS_2_ are covalently bonded, whereas multilayer MoS_2_ is bonded by the weak van der Waals force, and the space between adjacent layers is about 0.7 nm [6]. The band gap of MoS_2_ decreases with an increase of the number of layers due to quantum confinement [7]. Monolayer MoS_2_ has a direct band gap of 1.90 eV, which has the best luminescence properties. However, multilayer MoS_2_ has an indirect band gap of 1.29 eV [8,9]. As graphene-like material, the direct-bandgap semiconductor has excellent electrical and optical properties, so it can be used in the fabrication of photodetectors [10], transistors [11,12], sensors [13,14], and logic circuits.

There are two methods for the synthesis of monolayer MoS_2_: The top-down peeling method and the bottom-up growth method. It has been reported in the literature that the size of monolayer triangular MoS_2_ is 20–30 μm generally [15,16,17]. Novoselov et al. obtained monolayer MoS_2_ with the side length of 10 μm by the micromechanical stripping method, which is simple in process but low in yield and poor in repeatability [18]. Zeng et al. used the lithium ion intercalation method to grew monolayer MoS_2_ with the size of 20 μm [19]. This method has high stripping efficiency, large area and wide range, but the operation is complicated and the cost is high. Yan et al. used the hydrothermal method to obtain 4–6 nm thick MoS_2_ nano-sheets. The ammonium tetrathiomolybdate was used in a hydrothermal method, which has the advantages of simple operation, mild conditions, and low pollution, but it cannot control the synthesis of monolayer MoS_2_, and the crystallization quality is poor [20]. Zhan et al. and Lin et al. deposited Mo and MoO_3_ on the SiO_2_/Si substrate and the sapphire substrate, respectively. The deposited Mo and MoO_3_ are reacted with sulfur to grow the MoS_2_ film. The method is complicated and it is also difficult to control the deposition of uniform monolayer MoS_2_ film [21]. The current existing problem is that small-size monolayer MoS_2_ cannot meet the requirements for the application of optoelectronic devices. Therefore, the growth of the monolayer MoS_2_ with high quality, large size, and excellent performance has been became a research hotspot.

Here, the high quality, large-size monolayer MoS_2_ was grown on the Si substrate with 285-nm-thick SiO_2_ by atmospheric pressure chemical vapor deposition (APCVD) of sulfurized molybdenum trioxide. This growth method is simple, and it does not require vacuum treatment. The detailed growth parameters are: Temperature T = 720 °C, mass of the MoO_3_ powder M1 = 3 mg, mass of the S powder M2 = 100 mg, argon flow rate R = 35 sccm. Compared to previous literature reports, the grown MoS_2_ by APCVD is a uniform triangular monolayer with a side length of 100 μm. The specific sections are as follows: Section 2 introduces the specific growth experiment of MoS_2_, including substrate cleaning, heating process, and characterization methods. In Section 3, the growth experiments are studied in detail, and the optimal growth conditions are obtained. In Section 4, MoS_2_ sample under the optimal growth conditions is characterized by optical microscopy, AFM, XPS, Raman, and photoluminescence spectroscopy systematically. Section 5 is the conclusion of this paper.

## 2. Experimental Methods

**Figure 1:**
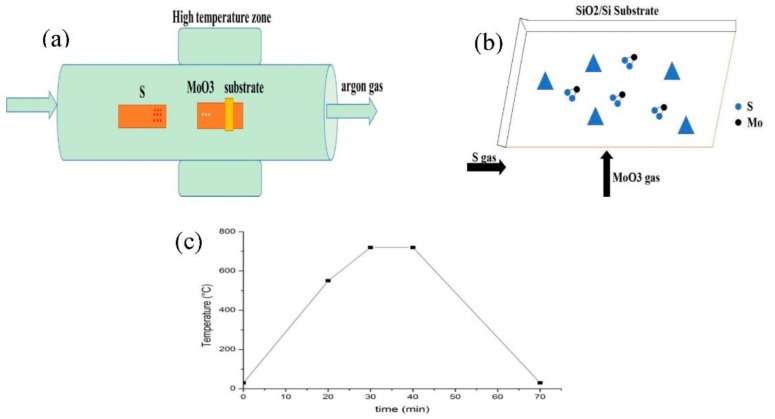
(**a**) Schematic illustration of the single temperature zone chemical vapor deposition (CVD) system; (**b**) schematic diagram of the MoS_2_ growth; (**c**) temperature change curve diagram of the SiO_2_/Si substrate.

The specific experiment steps are provided here. First, the 4-inch silicon wafer with a thickness of 285 nm SiO_2_ was cut into square small pieces with an area of 4–6 cm^2^ by a diamond pen. Then, the SiO_2_/Si substrates were placed into acetone, deionized water, absolute ethanol, and deionized water for ultrasonic cleaning in 15 min, 10 min, 15 min, and 10 min, respectively [22]. And these cleaned substrates were blown dry with the nitrogen gas gun. Subsequently, a certain mass of sulfur powder (purity, 99.5%, Alfa Aesar, Shanghai, China) and MoO_3_ powder (purity, 99.95%, Alfa Aesar, Shanghai, China) were weighed by the electronic analytical balance, and then put into two quartz boats, respectively. Next, the first quartz boat with S powder was placed in the low temperature zone of the tube furnace, and then the second quartz boat with MoO_3_ powder and SiO_2_/Si substrate was placed in the middle position of the tube furnace, wherein the cleaned SiO_2_/Si substrate was placed face down on the downstream from the MoO_3_ powder for 5 cm, as shown in Figure 1a. Afterwards, the argon gas (purity, 99.999%) was inserted into the tube furnace in order to purge the air in the tube furnace, a flow rate of 300 sccm (1 sccm = 1 mL/min) argon gas was then introduced in the tube furnace for 5 min before the heating process. Figure 1b shows the growth mechanism of MoS_2_. During the heating process, argon gas with a flow rate of 35 sccm was continuously supplied as the carrier gas. It can be observed from Figure 1c that the temperature of the SiO_2_/Si substrate rises from room temperature to 550 °C in 20 min initially, then rises to 720 °C in 10 min, and maintains the growth temperature 720 °C in 10 min, and then cools to room temperature naturally. Finally, the samples were taken out and some characterizations on the grown MoS_2_ samples were executed.

To characterize the surface morphology of grown MoS_2_, the new generation of high-resolution Raman spectrometer LabRam HR Evolution produced by HORIBA Jobin Yvon, was used. First, the MoS_2_ sample was placed on the stage of the microscope, then the Hg lamp emit laser light to the MoS_2_ sample. Subsequently, the scattered light from the monolayer MoS_2_ was collected and removed by the coupled optical path and the Rayleigh filter, respectively. Finally, the light was be converted into electrical signal by the charge coupled device (CCD), which is a special semiconductor device with many photosensitive elements in the LabRam HR Evolution with the single grating. The specific test conditions were: Spectral range of 300–450 cm^−1^; PL of 600–800 nm; spectral resolution ≤ 0.65 cm^−1^; spatial resolution was Lateral ≤ 1 μm, longitudinal ≤ 2 μm; Raman spectrum of 10 mW; PL spectrum of 25 mW; the scan time of 10 s; and the accumulation time of 3 s. The Raman and photoluminescence characterization were measured by using the LabRam HR Evolution with 532 nm laser and 50 × objective [23,24,25]. At the same time, in order to master the growth mechanism of monolayer MoS_2_, the X-ray photoelectron spectroscopy and Atomic Force Microscope, were also used.

## 3. The Experimental Results and Discussion

### 3.1. Effects of the Growth Temperature on MoS_2_

**Figure 2:**
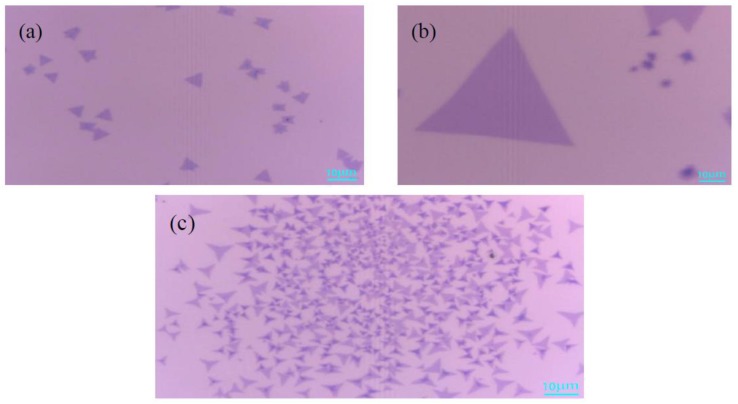
Optical microscope images of MoS_2_ grown at different temperatures (**a**) 670 °C; (**b**) 720 °C; (**c**) 770 °C. Scale bar: 10 μm.

Figure 2a is an optical micrograph of MoS_2_ grown at 670 °C. A small amount of triangular monolayer MoS_2_ on the substrate, 8–10 μm in size, was observed. The temperature was lower, the MoO_3_ powder evaporated slowly, and most of the MoO_3_ was carried away by the argon gas, which resulted in a small amount of MoS_2_ deposit on the substrate. Figure 2b shows a very regular triangular MoS_2_, and that the single crystal size can reach up to 40 μm, which indicates that the gas concentration of MoS_2_ is sufficient and the nucleation density of MoS_2_ increases when the growth temperature is 720 °C. In Figure 2c, the substrate exists as both triangular MoS_2_ and some body materials, which is due to the temperature being too high and the MoO_3_ powder evaporating quickly, thereby forming a mixture of MoO_3_ and MoS_2_. The above analysis indicates that 720 °C is the ideal temperature for growth of the monolayer MoS_2_.

### 3.2. Effects of the Mass of MoO_3_ Powder on MoS_2_

**Figure 3:**
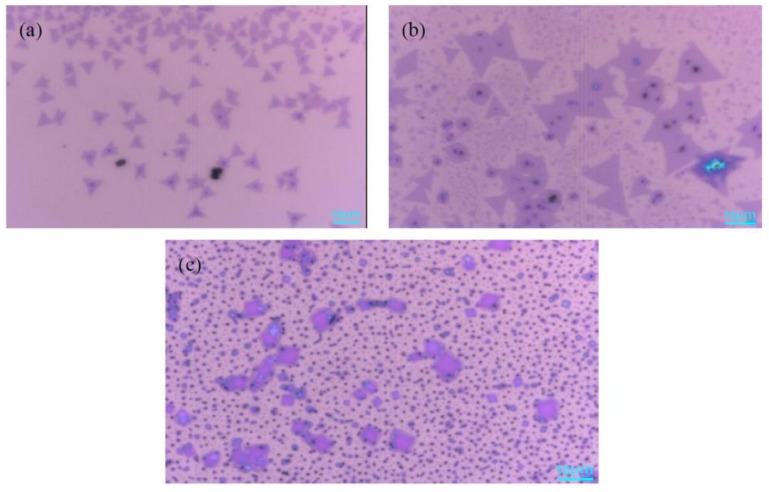
Optical microscope images of MoS_2_ with the different MoO_3_ mass (**a**) 1 mg; (**b**) 3 mg; (**c**) 5 mg. Scale bar: 10 μm.

The mass of MoO_3_ has a direct impact on the growth of MoS_2_ crystals. Under the premise that other growth conditions are consistent, 1 mg, 3 mg, and 5 mg of the MoO_3_ powder were weighed during the experiment, respectively. Figure 3a is an optical microscope image of MoS_2_ when the mass of MoO_3_ is 1 mg. It can be found that the size of the triangular single crystal MoS_2_ is about 10 μm, the surface of the sample is clean, and the contrast between the sample and the substrate is obvious. This is because the amount of MoO_3_ was small, which lead to low nucleation density. Figure 3b shows that the size of single crystal MoS_2_ can be as large as 30 μm, and the morphology of the sample was better, which reveals that the Mo:S ratio is >1:2 and the gas concentration of MoS_2_ is sufficient in this area. Meanwhile, the nucleation density is high. In Figure 3c, there is no obvious triangular single crystal MoS_2_, and the surface of SiO_2_/Si substrate is covered with fine black particles when the amount of MoO_3_ is increased to 5 mg. The reason is that the excessive amount of molybdenum source, which resulted in a large nucleation density and MoS_2,_ grew toward the bulk material. From the above comparison, it can be found that the optimum mass of MoO_3_ is 3 mg.

### 3.3. Effects of the Mass of S Powder on MoS_2_

**Figure 4:**
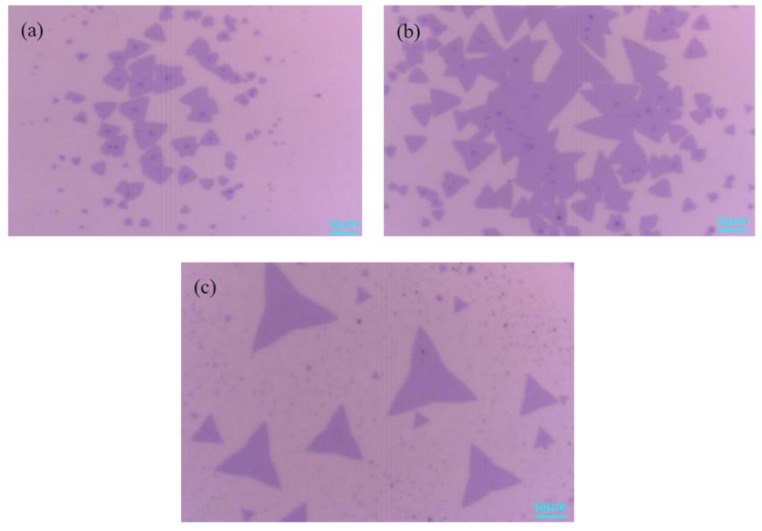
Optical microscope images of MoS_2_ with the different mass of S powder (**a**) 50 mg; (**b**) 100 mg; (**c**) 150 mg. Scale bar: 10 μm.

In Figure 4a, when the mass of S powder was 50 mg, some small triangles and black dots were formed on the substrate, the ratio of Mo:S was <1:2. The sulfur powder was too little to provide enough S gas to react with MoO_3_, which caused the MoO_3_ to be deposited on the substrate in the form of particles. Figure 4b shows that the shape of MoS_2_ is very regular, and the size of MoS_2_ can reach up to 40 μm, which reveals that the nucleation density is high and the gas concentration of MoS_2_ is sufficient in this area. Meanwhile, when the Mo:S ratio was >1:2, it increased the size of MoS_2_. When the mass of sulfur powder was increased to 150 mg, the shape of MoS_2_ in Figure 4c was the same as that in Figure 4b. When the Mo:S ratio was >1:2, it produced lager triangles. It can be inferred that the mass of S powder should be excessive during the experiment, which is beneficial to the growth of monolayer MoS_2_. The optimum mass of S powder is 100 mg according to the above results.

### 3.4. Effects of the Argon Flow Rate on MoS_2_

The high purity argon gas had two functions during the experiment. The first was to prevent impure gas from entering the tube furnace so that the reaction of sulfurized molybdenum trioxide would be carried out in the argon gas. The second was to transport S gas to the high temperature zone to react with the MoO_3_ gas. Finally, the MoS_2_ was transferred on the surface of substrate.

**Figure 5:**
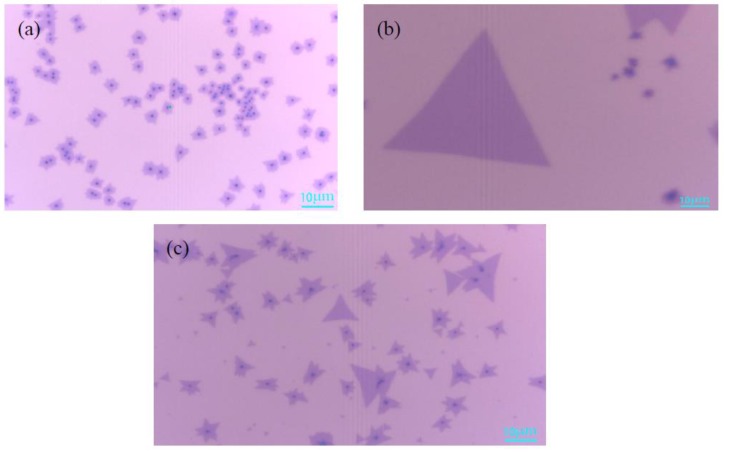
Optical microscope images of MoS_2_ with different argon flow rate (**a**) 20 sccm; (**b**) 35 sccm; (**c**) 50 sccm. Scale bar: 10 μm.

Figure 5a shows a mixture of MoO_2_ and MoS_2_ on the substrate. Owing to the sufficient S, gas could not be transported to the top of the MoO_3_, and the synthesis efficiency of MoS_2_ gas was relatively low, which limited the growth response of MoS_2_ when the argon flow rate is 20 sccm. In Figure 5b, it is observed that the shape of the MoS_2_ is triangular, and the MoS_2_ single crystal has a size of 60 μm when the argon gas flow rate is 35 sccm. The suitable argon flow rate caused the reaction of sulfurized molybdenum trioxide more thoroughly, which can make the gas concentration of MoS_2_ higher. In Figure 5c, the size of the MoS_2_ single crystal is greatly reduced, and the number of monolayer MoS_2_ is also sparse. It can be inferred that the surface of SiO_2_/Si substrate is not conducive to the nucleation and growth of monolayer MoS_2_ when the gas flow is 50 sccm. When the flow rate of argon gas becomes larger, the gas concentration and the nucleation density of MoS_2_ become smaller, resulting in the size of the triangular MoS_2_ becomes smaller. In summary, the optimal argon flow rate is 35 sccm.

According to the above analysis, the optimal growth conditions for monolayer MoS_2_ were obtained by atmospheric pressure chemical vapor deposition. The optimal growth parameters were: 720 °C temperature, 3 mg MoO_3_ powder, 100 mg S powder, and 35 sccm argon gas. At the same time, the Atomic Force Microscope, Raman spectroscopic, and X-ray photoelectron spectrum were used to analyze the prepared monolayer MoS_2_ sample under optimal growth conditions.

## 4. The Characterization of Monolayer MoS_2_ under Optimal Growth Conditions

**Figure 6:**
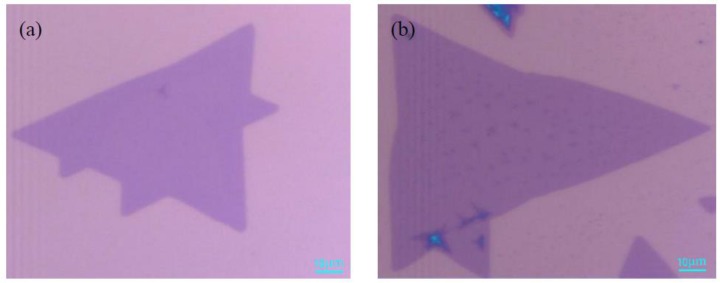
Optical microscope images of two different positions of the same sample (**a**) 80 μm; (**b**) 100 μm. Scale bar: 10 μm.

In Figure 6, it is observed that the grown MoS_2_ is a triangle with a size of 100 μm, which is much larger than the peeled off sample micromechanically. When the growth of MoS_2_ is under optimal growth conditions, the gas concentration of MoS_2_ is the largest and the nucleation density reaches a maximum. Moreover, the surface of the grown MoS_2_ is uniform, and the contrast between the sample and SiO_2_/Si substrate is obvious. Therefore, the grown MoS_2_ by optimal conditions can be judged to be a single-layer initially.

**Figure 7:**
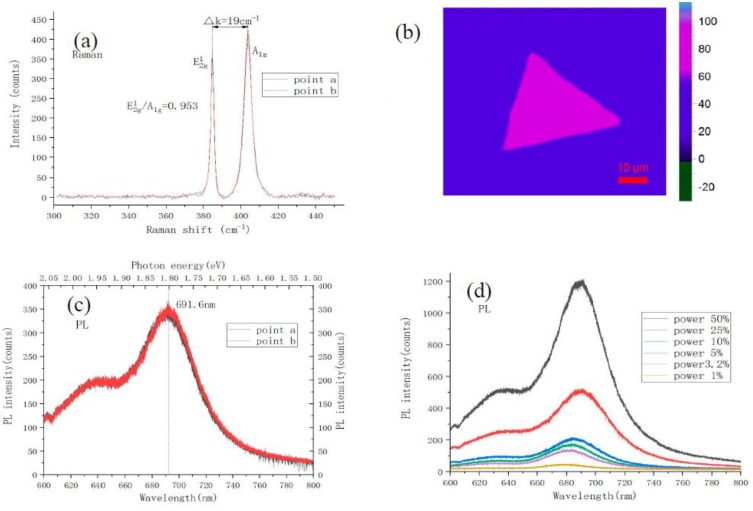
Raman and photoluminescence spectrum of the monolayer MoS_2_. (**a**) is the Raman spectrum of MoS_2_ corresponding to Figure 6, wherein the black line corresponds to Figure 6a, and the red line corresponds to Figure 6b; (**b**) is the Raman mapping of A_1g_; (**c**) shows the MoS_2_ photoluminescence spectrum corresponding to Figure 6. The black line corresponds to Figure 6a, and the red line corresponds to Figure 6b; (**d**) is the power-dependant PL spectrum under ambient condition.

Raman spectrum has become an effective method for the detection and identification of films prepared by chemical vapor deposition (CVD). The optical properties of monolayer MoS_2,_ obtained under optimal growth conditions, are characterized by Raman and PL spectroscopies. These spectral measurement conditions were in a super-clean lab with constant temperature and humidity. Figure 7a shows two obvious characteristic peaks in the Raman spectrum of MoS_2_, E^1^_2g_: 384.6 cm^−1^, A_1g_: 403.6 cm^−1^, which is fitted by the Gaussian-Lorenze curve. The E^1^_2g_ peak corresponds to the vibration of sulfur atoms in the horizontal plane, and A_1g_ peak represents the vibration of sulfur atoms in the vertical horizontal plane direction. Due to the gradual increase of the van der Waals force, the E^1^_2g_ characteristic peak was blue-shifted, and the A_1g_ characteristic peak was red-shifted when the number of layers was reduced [26]. Therefore, the layer number of MoS_2_ sample can be determined based on the wavenumber difference of two characteristic peaks A_1g_ and E^1^_2g_ modes. It can be seen from the above two peak positions in Figure 7a that the wavenumber difference is about 19 cm^−1^, and the intensity ratio of E^1^_2g_/A_1g_ ≈ 0.95, which indicates that the grown MoS_2_ sample is a monolayer. Figure 7b is a triangular MoS_2_ single crystal with Raman mapping image of the A_1g_ peak intensity (4000 points total), which is randomly selected on the grown MoS_2_ sample. It can be seen from Figure 7b that the thickness of triangular MoS_2_ is uniformly high. To the best of our knowledge, when MoS_2_ transforms from bulk material into monolayer material, the band gap changes from indirect band gap to direct band gap, and the fluorescence efficiency is enhanced significantly. Figure 7c shows the PL spectrum fitted by the Gaussian-Lorenze curve, the two main exciton peaks of the monolayer MoS_2_ luminescence are located at 642 nm (B excitons) and 691 nm (A excitons), respectively. The neutral excitons of A and B arise from valence band to conduction band transitions within the direct band gap of monolayer MoS_2,_ directly. The relative contribution of excitons and triangles determines the peak position of PL spectrum. The shape of PL spectrum is consistent with the previous reported result of monolayer MoS_2_ obtained by mechanically exfoliated, CVD, physical vapor deposition (PVD), and so on [27,28,29]. Due to strain during thermal growth and different test environments, the peak position of A and B exciton results in a small fluctuation shift, which is within the allowable range [30]. According to the conversion relationship between wavelength and electron volt, the photon energy is inversely proportional to the wavelength:

(1) $$E=h\times H=\frac{h}{k}\times \frac{C}{\lambda }=\frac{1243}{\lambda }$$

In the formula (1), the symbol *E*, *h*, *C*, *λ* and *k* refer to the energy, Planck constant, light speed, wavelength and constant, respectively. Their units respectively are eV, J·s, nm/s, nm and J/eV.

The strongest luminescence peak is at 691.6 nm, the transition energy level is about 1.80 eV in Figure 7c. It is known that the direct band-gap of monolayer MoS_2_ is 1.8–1.9 eV, which can further prove that the sample is a monolayer MoS_2_. Figure 7d is the power-dependent PL spectrum of monolayer MoS_2_ under ambient condition, it can be seen that the PL intensity increases as laser power increases, and the relative positions of the A and B neutral excitons in the PL spectrum take place red-shifting, when the laser power increases due to the n-type doping of SiO_2_/Si substrate.

**Figure 8:**
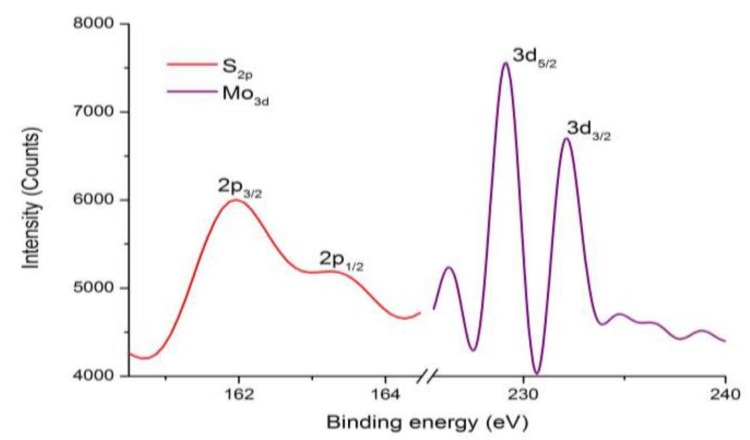
XPS spectra of Mo_3d_ and S_2p_ for the monolayer MoS_2._

In order to obtain the binding energy of grown MoS_2_, the X-ray photoelectron spectroscopy (XPS) of Theta 300 XPS system, produced by Thermo Fisher, was adopted. The specific test conditions were: Source Gun Type: Al K Alpha; Spot Size: 650 μm; Lens Mode: Standard; Analyser Mode: Pass Energy 30.0 eV of CAE; Energy Step Size: 0.100 eV; Number of Energy Steps: 181, the number of scans in S_2p_ and Mo_3d_ is 8 and 7, respectively. The X-ray photoelectron spectrum image of the MoS_2_ sample is shown in Figure 8, it can be observed that the MoS_2_ sample have Mo^4+^ and S^2−^ signals, which is consistent with the results reported in the literature [31]. The 3d orbital peaks of Mo are at 229.17 eV and 232.17 eV, corresponding to 3d_5/2_ and 3d_3/2_ orbitals respectively. The 2p orbital peaks of S are at 161.97 eV and 163.27 eV, corresponding to 2p_3/2_ and 2p_1/2_ orbitals respectively. At the same time, the atomic percentages of Mo_3d_ and S_2p_ elements are 5.42% and 11.36%, respectively, which indicates that the ratio of the atomic numbers of Mo and S is approximately 1:2. It indicates the presence of monolayer MoS_2_.

**Figure 9:**
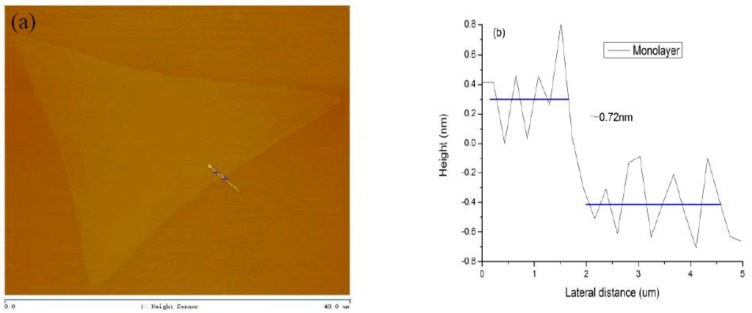
(**a**) Two-dimensional AFM topography image of the monolayer MoS_2_; (**b**) Height profile of the monolayer MoS_2._

Figure 9a shows that the surface roughness of MoS_2_ sample is extremely low, the contrast and height of the sample surface are not changed obviously, which indicates the formation of monolayer MoS_2_ [32]. The height of the MoS_2_ sample in Figure 9b is around 0.72 nm. Therefore, it further evidences that the grown MoS_2_ is a single layer.

## 5. Conclusions

The large-size and high-quality monolayer MoS_2_ is grown on the Si substrate with a 285-nm-thick oxide layer by APCVD. The effects of MoO_3_ powder, S powder, temperature and argon flow rate on the growth of MoS_2_ are analyzed systematically. The optimum growth condition parameters for monolayer MoS_2_ are: 720 °C temperature, 3 mg MoO_3_ powder, 100 mg S powder and 35 sccm argon gas. The above results can effectively help us to understand the morphology and optical properties of monolayer MoS_2_. The optical microscopy, XPS, AFM, Raman, and photoluminescence spectroscopy are used to characterize the grown monolayer MoS_2_ under optimal condition. The results show that the grown MoS_2_ sample is a single layer with the size of 100μm, which can pave the way for the applications of photodetectors and transistors.
